# Green Corn

**Published:** 1871-10

**Authors:** 


### GREEN CORN.

Thrifty farmers arrange to have good, sweet, tender corn for their tables until late in the fall of the year; its tenderness, nutritiveness, and digestibility are greatly improved by running a sharp knife along the centre of each row, before eating it, thus dividing every grain in half. Dangerous bowel complaints are often induced by chewing green corn imperfectly. Every grain should be ground by the teeth into a pulp, before it is swallowed. For sedentary persons one ear of corn is enough for one meal.

